# Supplementary material for: Deconvoluting the Optical Response of Biocompatible Photonic Pigments
Source: Angew Chem Int Ed Engl. 2022 Jul 13;61(34):e202206562. doi: 10.1002/anie.202206562 (PMC9542403; doi:10.1002/anie.202206562)
Supplement: Supplementary file 1 — Supporting Information [file ANIE-61-0-s001.pdf]

Angewandte Chemie
Eine Zeitschrift der Gesellschaft Deutscher Chemiker

Supporting Information

Deconvoluting the Optical Response of Biocompatible Photonic Pigments

Z. Wang, C. L. C. Chan, J. S. Haataja, L. Schertel, R. Li, G. T. van de Kerkhof, O. A. Scherman, R. M. Parker, S. Vignolini**

Supporting Information

Deconvoluting the Optical Response of Biocompatible Photonic Pigments

Zhen Wang,[1] *Chun Lam Clement Chan,*[1] *Johannes S. Haataja,*[1] *Lukas Schertel,*[1,2] *Ruiting Li,*[1] *Gea T. van de Kerkhof,*[1] *Oren A. Scherman,*[1] *Richard M. Parker,*[1]* *Silvia Vignolini*[1]*

[1] Melville Laboratory for Polymer Synthesis, Yusuf Hamied Department of Chemistry, University of Cambridge, Lensfield Road, Cambridge, CB2 1EW, United Kingdom

[2] Department of Physics, University of Fribourg, Chemin du Musée 3, 1700 Fribourg, Switzerland

*E-mail: sv319@cam.ac.uk; rmp53@cam.ac.uk.

Section S1: Additional synthetic and sample preparation methodology

Materials

5-Norbornene-2-methanol (NB-OH, 98%, mixture of *endo* and *exo*), tin (II) 2-ethylhexanoate ($Sn(Oct)_2$, 92.5-100.0%), polyethylene glycol methyl ether (PEG-OH, M_n~2000 g mol^{-1}), *exo*-5-norbornenecarboxylic acid (NB-COOH, 97%), *N*,*N*'-dicyclohexylcarbodiimide (DCC, 99%), poly(vinyl alcohol) (PVA, M_w = 13,000-23,000 g mol^{-1}, 87-89% hydrolyzed), second generation Grubbs catalyst (M2a(C848: benzylidene[1,3-bis(2,4,6-trimethylphenyl)-2-imidazolidinylidene]dichloro(tricyclo-hexylphosphine)ruthenium) were purchased from Merck Life Science UK Ltd. Pentane (99%) was obtained from Scientific Laboratory Supplies Ltd. ε-Caprolactone (CL, 99%, ACROS Organics) and dichloromethane (DCM, 99.8%, extra dry over molecular sieve, Acros Organics), 3-bromopyridine (99%, Acros Organics) were provided by Fisher Scientific Ltd. CL was dried with activated molecular sieve before use. 4-(Dimethylamino)pyridine (DMAP, ≥99%) was purchased from Alfa Aesar. Dichloromethane (99%, DCM), methanol (99%, MeOH), and toluene (99%) were provided by Fisher Scientific UK Ltd. Deionized water (resistivity >18 MΩ cm^{-1}) was purified using a Millipore Milli-Q Synergy system. Unless stated explicitly, all chemicals were used without further purification.

Synthesis of norbornene-poly(ε-caprolactone) macromonomer (NB-PCL)

NB-PCL macromonomer was synthesized via ring-opening polymerization of CL. 20.8 g CL (182.0 mmol, 22 eq.), 1.0 g NB-OH (8.3 mmol, 1 eq.) and 167.7 mg of $Sn(Oct)_2$ (0.4 mmol, 0.05 eq.) were added in a heat-dried Schlenk tube equipped with a stirrer bar. The tube was sealed and stirred under N_2 for 2 h at 100 °C. After cooling to room temperature, the reaction mixture was diluted with DCM and precipitated into cold MeOH. The solid product was redissolved in DCM and the precipitation repeated two more times with MeOH. The resultant polymer was dried overnight in a vacuum oven at 50 °C (M_n = 3.1 kg mol^{-1}).

Synthesis of norbornene-polyethylene glycol macromonomer (NB-PEG)

NB-PEG macromonomer was synthesized via Steglich esterification. 20.0 g PEG-OH (10.0 mmol, 1 eq.), 2.8 g NB-COOH (20.0 mmol, 2 eq.) and dry DCM (150 mL) were added in a Schlenk tube equipped with a stirrer bar. The tube was sealed and stirred at room temperature under N_2 protection for 20 min. DCC (5.0 g, 24.0 mmol, 2.4 eq.) was then added under nitrogen. The mixture was subsequently cooled to 0 °C and stirred for 30 min. A solution of DMAP (125 mg, 1.0 mmol, 0.1 eq.) in DCM (5 mL) was added in the solution, which was

then stirred for 24 h at room temperature. The reaction mixture was filtered to remove any precipitate and the filtrate was added to a 10-fold excess of cool diethyl ether to precipitate the product as a white solid. Two more precipitations in diethyl ether were carried out to remove excess NB-COOH. The white solid was dried in a vacuum oven at 40 °C for 24 h (M_n = 2.4 kg mol^{-1}).

Synthesis of [(H_2IMes)(3-Br-py)$_2$(Cl)$_2$Ru=CHPh] (Third generation Grubbs catalyst, G3)

3-Bromopyridine (0.34 mL, 3.5 mmol, 10 eq.) was added to G2 catalyst (0.3 g, 0.35 mmol, 1 eq.) in a 20 mL vial with a snap cap; no additional solvent is required. The reaction was stirred at room temperature in air for 10 min during which a color change from red to bright green was observed. Pentane (20 mL) was added to the green solution, leading to the precipitation of a green solid. The vial was subsequently capped under air and cooled to ~ -20 °C overnight (freezer). The green precipitate was collected using vacuum filtration, washed with 4 × 10 mL of pentane, and dried under vacuum to afford the product as a green powder.

Synthesis of poly(NB-PCL)-b-poly(NB-PEG) block copolymers via ring opening metathesis polymerization (ROMP). In a typical experiment, 315 mg of NB-PCL (3.1 kg mol^{-1}, 0.01 mmol) and 220 mg of NB-PEG (2.4 kg mol^{-1}, 0.009 mmol) were added separately into vials, which were degassed and backfilled with nitrogen. Anhydrous dichloromethane (DCM), which was degassed via three freeze-pump-thaw cycles, was added to the two vials resulting in macromonomer solutions between 0.1-0.2 M. At room temperature, the polymerization of NB-PCL was initiated by adding the precise volume of G3 catalyst as a solution in DCM solution (1.8 mg mL^{-1}), using a syringe pump. After 3 min, the solution of the second macromonomer (NB-PEG) was injected into the reaction mixture. This solution was allowed to stir at room temperature for an additional 2 h. The reaction was then quenched with ethyl vinyl ether, diluted with DCM and precipitated into cold methanol. The produced BBCP was dried overnight in a vacuum oven at 50 °C. The desired degree of polymerization was achieved by maintaining the same quantities of macromonomers and varying the volume of G3 catalyst solution. The amount of catalyst added for each polymer and the resultant M_n are reported in **Table S2**.

Preparation of BBCP microemulsions via a microfluidic device. Monodisperse microdroplets were generated within a hydrophilic, etched-glass microfluidic device (Dolomite #3000158, Droplet Junction Chip with 100 μm etch depth). The discontinuous phase was prepared by dissolving the block copolymers (CLEG1-4) in toluene (30 mg mL^{-1}) For polymer-

macromonomer mixtures, the solutions were prepared by dissolving 6-36 mg NB-PCL into 1 mL of CLEG1 or 4 solution (30 mg mL^{-1}) , i.e. a macromonomer loading of 20-120 wt.% relative to the BBCP. The continuous phase contained polyvinyl alcohol (PVA, 200 mg) as a stabilizer, dissolved in Milli-Q water (10 mL). To form a toluene-in-water microemulsion, the aqueous PVA solution (2 w/v%) and the BBCP solution in toluene were injected into the microfluidic device using two syringe pumps with flow rates of 500 and 200 μL h^{-1}, respectively. At the junction, the toluene-in-water emulsions were formed by the shear forces, leading to stable droplets with diameter, Ø ≈ 170 μm. The droplets (ca. 23 μL) were collected into a vial (7 mL) filled with PVA solution (ca. 2 mL) for further solvent evaporation.

Preparation of BBCPs photonic microparticles. For CLEG1, vials containing polymer-toluene droplets were placed in a pre-heated water bath at a defined temperature (30, 40, 50, 60, 70 and 80 °C). For investigation of the effect of molecular weight, droplets containing CLEG2-4 were evaporated at 50 °C. For polymer-macromonomer mixtures, the droplets were evaporated in 60, 65, 70, and 75 °C. In all cases, discrete polymer microparticles were formed after loss of toluene. Once dry, the microparticles dispersed in water (typically Ø ≈ 70 μm) were washed with Milli-Q water to remove residual PVA surfactant.

Section S2: Additional characterization methods

Optical microscopy **and** ***micro-spectroscopy*** were performed on a customized Zeiss Axio Scope A1 microscope fitted with a CCD camera (Eye IDS, UI-3580LE-C-HQ, calibrated with a white diffuser) using a halogen lamp (Zeiss HAL100) as a light source. To perform micro-spectroscopy, the microscope was coupled to a spectrometer (Avantes, AvaSpecHS2048) using an optical fiber (Avantes, FC-UVIR200-2, 200 μm core size). The reflectance spectra were normalized against a white diffuser (Labsphere USRS-99-010). The BBCP microspheres dispersed in water were analyzed using a water immersion objective (Zeiss, W N-Achroplan 40x/0.75 M27 (FWD=2.1mm)). The collected spectra are analyzed based on the methodology presented in **Section S3**, Supporting Information. *Optical photographs* were taken using Canon EF 50mm f/1.8 II lens with Kenko 12 mm extension tube mounted to Canon EOS-1D Mark III body.

***Scanning electron microscope* (SEM)** images were collected with a Mira3 system (TESCAN) operated at 3 kV and a working distance of 3 - 6 mm. The samples were mounted on aluminum stubs using conductive carbon tape and coated with Pt (10 nm) using a sputter coater (Quorum Q150T ES). The samples were obtained by lyophilizing the microparticle suspensions. The samples were fractured mechanically using a microspatula to expose the cross-section.

^{1}H NMR spectroscopy was recorded in $CDCl_3$ using a Bruker 400 MHz Avance III HD Spectrometer.

Cryogenic Scanning electron microscopy (Cryo-SEM) was performed at the Cambridge Advanced Imaging Centre using an FEI Verios 460 scanning electron microscope, equipped with a Quorum cryo-transfer system (PP3010T). Samples were washed with Milli-Q water to remove surfactant. A small amount of this suspension of microspheres in water was then placed on top of a freezing rivet mounted on the specimen holder, forming a spherical droplet. The specimen holder with sample was then plunge frozen in liquid ethane and transferred to a specimen preparation chamber cooled to −140 °C. The droplets were then freeze-fractured, exposing the internal structure of the microspheres. Samples were then sublimed at −90 °C for 10 minutes and subsequently sputter-coated with platinum. Imaging was performed at 2.00 kV acceleration voltage with a 13 pA probe current.

Gel permeation chromatography (GPC) was used to determine the molecular weights of the macromonomers and the block copolymers. GPC was carried out in DMF (eluent flow rate of 1.0 mL min^{-1}) using three PLgel 10 μm mixed-B LS columns (Polymer Laboratories). For macromonomers (NB-PCL), the molecular weights were obtained relative to a series of polystyrene standards. For block copolymers, their absolute molecular weights were determined by combining GPC and light scattering technique, which is widely used to measure polymers with a non-linear architecture. The GPC was connected in series with a DAWN HELEOS multi-angle light scattering (MALS) detector and a refractive index (RI) detector. The d*n*/d*c* values were first measured by injecting 5 concentrations of polymer solutions directly into RI detector without passing through the columns. From the d*n*/d*c* values, the absolute molecular weights were obtained by analysis of the signals obtained from the MALS detector.

Optical Microscopy with an inverted microscope (Zeiss Axio Vert.A1) connected with a CCD camera (Pixelink PL-D725CU-T) was used to monitor the preparation process of the microdroplets.

Dynamic Light Scattering (DLS) was performed with a glass cuvette with round aperture on a ZETASIZER NANO-ZS with 633nm He-Ne LASER (Malvern Panalytical). The polymer-toluene solutions (1 mL, 5 mg mL^{-1}) were added in the cuvette and equilibrated for 5 min at a specific temperature (22, 30, 40, 45, or 50 °C) and water (200 μL) at the same temperature was added to initiate the measurements. Before each measurement, the mixture was gently shaken to obtain a uniform micelle suspension. Measurement angle, position, and the attenuator were fixed at 173°, 6.5 mm, and 11, respectively. Constant refractive indices for the material (1.470) and dispersant (1.496) were used through the measurements. At different temperatures, different viscosities were used for the dispersant phase (toluene), as defined in the Zetasizer software. Specifically, the viscosities at 22, 30, 40, 45, and 50 °C are 0.5762, 0.5259, 0.4723, 0.4488, and 0.4272 cP, respectively.

Dynamic Interfacial tension (IFT) was measured in a Contact Angle and Surface Tensiometer (First Ten Ångstroms 100) via pendant drop shape analysis. Typically, a drop of BBCP toluene solution (30 mg mL^{-1}) was suspended in water, which formed an inverted droplet because of the lower density of toluene compared to water. A series of images were recorded by a camera at an interval of 2 s over 5 min. By fitting the droplet shape with the associated software, the values of the interfacial tension were obtained.

Section S3: Spectra and image analysis

The reflectance spectrum of 50 photonic microparticles was collected with the setup in **Methods**. The mean of these spectra was used to plot the representative spectrum in **Figure 4**, and also **Figure S10, S24, S28**, **S29**, and **S30**. The colored band represents the standard deviation of these 50 spectra. The optical spectra were analyzed by first correcting for the baseline. The baseline was taken as the spectrum of a water layer on a glass slide, which was deducted from each spectrum. The position of the peak at large wavelength was extracted by peak-picking. The position of the first structural peak was obtained by fitting the spectra with Lorentzian distributions. For spectra that appear as a bimodal distribution or a unimodal

distribution with a shoulder, the spectra were modelled as the sum of two Lorentzian distributions while spectra with only one peak (without a shoulder) were described by one Lorentzian distribution. The form of each Lorentzian curve was taken as

$$y = \frac{a}{\pi}\frac{c}{(x-b)^2+c^2} \qquad (1)$$

where the peak position is given by b. The presented positions for each sample were averages calculated using values extracted from 50 measurements, while the errors were calculated from the standard deviation.

For structural analysis, let $I(\boldsymbol{x})\colon \mathbb{R}^n \to (0,1)$ denote binarized 2D images and 3D volumes for *n=2* and *n=3*, respectively. The 2-point correlation function (structure factor) is then defined as

$$S_2(r) = \langle I(\boldsymbol{x})I(\boldsymbol{x}+\boldsymbol{r})\rangle \qquad (2)$$

where $r = |\boldsymbol{r}|$, and the angular brackets denote ensemble averages and the correlation length is defined as

$$\xi = argmin_r\, S_2(r)) \qquad (3)$$

For experimental data, SEM micrographs were segmented to *I(**x**)* using the trainable WEKA segmentation tool in ImageJ.[1] The correlation length was then calculated using fast Fourier techniques in Matlab, and the pore sizes were calculated using ImageJ particle analysis tools after removing noise from the images via the "remove outliers tool" in ImageJ.[2]

The extracted apparent pore size (**Figure S15a**) was analyzed using a Matlab code developed by Depriester and Kubler based on the Saltykov method.[3] Excluding outlying pore sizes with radii above 300 nm, a histogram (with 25 bins) of the real pore size was accordingly generated (**Figure S15b**). The bin centers and heights were subsequently fitted to a log-normal distribution with the form

$$\frac{1}{r\sigma\sqrt{2\pi}}e^{-\frac{(\log(r)-\mu)^2}{2\sigma^2}}$$

Thus, the histogram could be approximated as a continuous probability density function, of which the median e^{μ} can be taken as the average pore size $|r|$. The error in the average pore size was approximated from the distribution fitting by the confidence bound at a level of 95%. The full width at half maximum probability of the pore size distribution curves

$$e^{(\mu-\sigma^2)+\sqrt{2\sigma^2\ln 2}} - e^{(\mu-\sigma^2)-\sqrt{2\sigma^2\ln}}$$

was taken as the pore size distribution width (w_r).

Section S4: 3D simulation

Let $f(x) : \mathbb{R}^3 \rightarrow \mathbb{R}$ represent a continuous field. The structural simulations were carried using a phase-field crystal model approach suggested by Guttenberg *et al.*[4] The following equation of motion:

$$\frac{\partial f}{\partial t} = \nabla^2 \left[(\nabla^2 + 1)^2 f + \alpha(-f + f^3)\right] \qquad (4)$$

where $\alpha = 20$ is the undercooling parameter, and the mean density $\langle f \rangle = 2\phi_0 - 1$, $\phi_0 = 0.23$, was solved by using a spectral method to simulate the time evolution of the system.[5] The simulations were performed in a simulation box with N^3 grid points under periodic boundary conditions, and the scale of the structures were controlled by varying N between 500 to 700. The 3D surface models (**Figure 2a**, **c**, **e**, **g**) representing the photonic structures were then obtained by using a level-set scheme:

$$I(x) = \begin{cases} 1, & \text{if } f(x) \geq \rho_0 \\ 0, & \text{otherwise} \end{cases}$$

where ρ_0 is the threshold value, using UCSF Chimera,[6] and then imported to Lumerical FDTD solver as 5 µm × 5 µm × 5 µm volumes. The FDTD simulations were carried out using Lumerical version 2020a-r5 (Ansys Canada Ltd.), with non-periodic boundary conditions and perfect matching layer boundaries in *x,y*-directions and broad band source $\lambda \in [200, 800]$ nm in coming from the vertical direction. The refractive index for the material was set to $n = 1.50$ and $n = 1.33$ for the surrounding medium in all simulations. The reflectance was then measured with the monitor set 7 µm above the sample, behind the source, and spatially average over the detector area.

Section S5: Analytical model of scattering

Calculating the Mie-scattering cross section of a single sphere:

For a plane wave scattered by a spherical particle the scattering cross section is given by:[7]

$$\sigma_s = \frac{2\pi}{k^2} \sum_{l=1}^{\infty} (2l + 1)(|a_l|^2 + |b_l|^2)$$

with a_l and b_l the Mie coefficients calculated with the use of Bessel functions of first and second order.

Multiple light scattering in the diffusive regime

In the multiple scattering regime the transport mean free path l^*, as a microscopic property, can be directly related to the reflection R of a system via R=1-T (where T is transmission, assuming no absorption) with[8]

$$T \sim \frac{l^*}{L}$$

The mean free path connects to the scattering cross section via

$$l^* \sim \frac{1}{\rho_S \sigma_S}$$

Where ρ_S is the number density of scatters and σ_s is the scattering cross section. The scattering cross section can be expressed in terms of the form factor F(θ) (scattering properties of the single sphere) and the structure factor S(θ) (collective scattering of the sample)[7, 9]

$$\sigma_s = \frac{\pi}{k^2} \int_0^{\pi} F(\theta)\, S(\theta) \sin\theta \, d\theta$$

Calculating the structural correlation for a random packing of hard spheres:

For randomly packed hard spheres, one can calculate the structure factor $S(\theta)$ (collective scattering of the sample) using the Percus-Yevick approximation:[10]

$$S(q) = 1/(1 - n_\rho \tilde{c}(q))$$

Here q = 2k sin θ/2 with k = $2\pi/\lambda$ and λ the wavelength of the light in the surrounding medium. $\tilde{c}(q)$ can be calculated via the Ornstein-Zernike equation and $n_\rho = (6f)/\pi r^3$ is the number density that can be expressed via the volume fraction f and the scatterer radius r.

Section S6: Color saturation and purity

To better quantify how the pigments appear to the human eye, their coordinates in the CIELAB color space were calculated from the reflection spectra using a standard observer (CIE 1931 2°) and relative to a standard illuminant (Daylight D65).[11] As shown in **Figure S32a**, this chromaticity diagram has three coordinates representing lightness (L*, between 0 and 100), the position between red and green (a*, green when a*< 0, red when a*> 0), and the position between yellow and blue (b*, blue when b*< 0, yellow when b*> 0) of the color, which allows

for detecting perceptual color difference between any two colors. The coordinates are calculated according to an open-source Python package.[12]

The chroma (C*) is calculated according to:

$$C^* = \sqrt{(a^{*2} + b^{*2})}$$

which is conceptually close to saturation, representing the purity of the color.[13] The values from different photonic pigments prepared in different temperatures or with different BBCPs were normalized and compared in **Figure S32b**.

Section S7: DLS analysis

The amphilicity of **CLEG1**, as validated by interfacial tension measurements (see **Figure S12, Video S1**), can promote the formation of BBCP micelles, the presence of which can be monitored in real time by DLS. When **CLEG1** was dissolved in pure toluene, no micelles were detected, even at high BBCP concentrations (e.g., 30 mg mL^{-1}). However, once the homogenous BBCP solution was exposed to a water drop, subsequent diffusion into the toluene phase stimulated their formation. As reported in **Figure S13b**, when the experiment was carried out at 22°C, initially nothing was detected by DLS, but after 5 minutes, micelles started to form in the solution and their number increased rapidly as more water diffused into the toluene. In parallel, their size decreased as more micelles formed until a dynamic equilibrium was finally reached, in which the size and number of the micelles was relatively stable (**Figure S13b-c**). Notably, the dispersity of the micelle size sharpened during the early stages of this process and increased slightly as the micelles swelled (**Figure S13d**). The resulting narrow distribution in size supports the mechanism of micelles first forming upon emulsification of the BBCP toluene solution in water, and then geometrically packing upon subsequent toluene evaporation to yield the uniform porous structures observed in **Figure 1d**, with the BBCP shells of the micelles fusing upon final drying to form the extended polymer matrix. Repeating the DLS measurement at a higher temperature resulted in a smaller number of micelles, but of a larger size (**Figure S13b-c**), which is attributed to the increased solubility of water in toluene.[14] Importantly, this phenomenon can explain the increase in pore size and corresponding redshift in the color of the photonic pigments.

Section S8: References

[1] I. Arganda-Carreras, V. Kaynig, C. Rueden, K. W. Eliceiri, J. Schindelin, A. Cardona, H. Sebastian Seung, *Bioinformatics* **2017**, *33*, 2424-2426.

[2] J. Schindelin, I. Arganda-Carreras, E. Frise, V. Kaynig, M. Longair, T. Pietzsch, S. Preibisch, C. Rueden, S. Saalfeld, B. Schmid, J.-Y. Tinevez, D. J. White, V. Hartenstein, K. Eliceiri, P. Tomancak, A. Cardona, *Nat. Methods* **2012**, *9*, 676-682.

[3] D. Depriester, R. Kubler, *Image Anal. Stereol.* **2019**, *38*, 213-226.

[4] N. Guttenberg, N. Goldenfeld, J. Dantzig, *Phys. Rev. E* **2010**, *81*, 065301.

[5] S. B. Biner, *Programming phase-field modeling*, Springer, **2017**.

[6] E. F. Pettersen, T. D. Goddard, C. C. Huang, G. S. Couch, D. M. Greenblatt, E. C. Meng, T. E. Ferrin, *J. Comput. Chem.* **2004**, *25*, 1605-1612.

[7] C. F. Bohren, D. R. Huffman, *Absorption and scattering of light by small particles*, John Wiley & Sons, **2008**.

[8] M. Reufer, L. F. Rojas-Ochoa, S. Eiden, J. J. Sáenz, F. Scheffold, *Appl. Phys. Lett.* **2007**, *91*, 171904.

[9] S. Fraden, G. Maret, *Phys. Rev. Lett.* **1990**, *65*, 512-515.

[10] J. K. Percus, G. J. Yevick, *Phys. Rev.* **1958**, *110*, 1-13.

[11] T. B. S. Institution, in *Colorimetry - Part 4: CIE 1976 L*a*b* colour space.*, **2019**.

[12] T. Mansencal, M. Mauderer, M. Parsons, N. Shaw, K. Wheatley, S. Cooper, J. D. Vandenberg, L. Canavan, K. Crowson, O. Lev, K. Leinweber, S. Sharma, T. J. Sobotka, D. Moritz, M. Pppp, C. Rane, P. Eswaramoorthy, J. Mertic, B. Pearlstine, M. Leonhardt, O. Niemitalo, M. Szymanski, M. Schambach, S. Huang, M. Wei, N. Joywardhan, O. Wagih, P. Redman, J. Goldstone, S. Hill, J. Smith, F. Savoir, G. Saxena, S. Chopra, I. Sibiryakov, T. Gates, G. Pal, N. Tessore, A. Pierre, *Colour 0.4.1*, Zenodo, **2022**.

[13] K. McLaren, *J. Soc. Dyers. Colour.* **1976**, *92*, 338-341.

[14] M. Goral, B. Wisniewska-Goclowska, A. Skrzecz, I. Owczarek, K. Blazej, M.-C. Haulait-Pirson, G. T. Hefter, F. Kapuku, Z. Maczynska, A. Szafranski, C. L. Young, *J. Phys. Chem. Ref. Data* **2005**, *34*, 1399-1487.

Section S9: Additional tables

Table S1. Number average molecular weight (M_n), weight average molecular weight (M_w), and polydispersity (PDI) of PCL macromonomer (NB-PCL) prepared with different polymerization times. The molecular weights were determined relative to a series of polystyrene standards.

Sample name	Polymerization duration (h)	M_n (g mol^{-1})	M_w (g mol^{-1})	PDI
NB-PCL-0.5 h	0.5	1553	1800	1.16
NB-PCL-1.0 h	1.0	2643	2981	1.13
NB-PCL-1.5 h	1.5	3241	3723	1.15
NB-PCL-2.0 h	2.0	3610	4226	1.17
NB-PCL-2.5 h	2.5	3836	4608	1.20
NB-PCL-3.0 h	3.0	3846	4831	1.26

Table S2. Summary of experimental and characterization data for the four BBCPs: CLEG1, CLEG2, CLEG3, and CLEG4.

BBCP name	G3 solution (µL)	DP_1 (NB-PCL)	DP_2 (NB-PEG)	M_n (kg mol^{-1})	M_w (kg mol^{-1})	PDI	d_n/d_c
CLEG1	625	113	98	589	705	1.20	0.0496 ± 0.0011
CLEG2	769	97	87	513	559	1.09	0.0484 ± 0.0009
CLEG3	526	151	137	800	953	1.19	0.0490 ± 0.0008
CLEG4	455	206	187	1095	1307	1.19	0.0485 ± 0.0006

Section S10: Additional video

Video S1: When the toluene solution of NB-PEG (30 mg mL^{-1}) was added in water by the pendant drop method during the interfacial tension measurements, small droplets were formed in the pendant toluene droplet as water was absorbed into the droplet.

Section S11: Additional figures

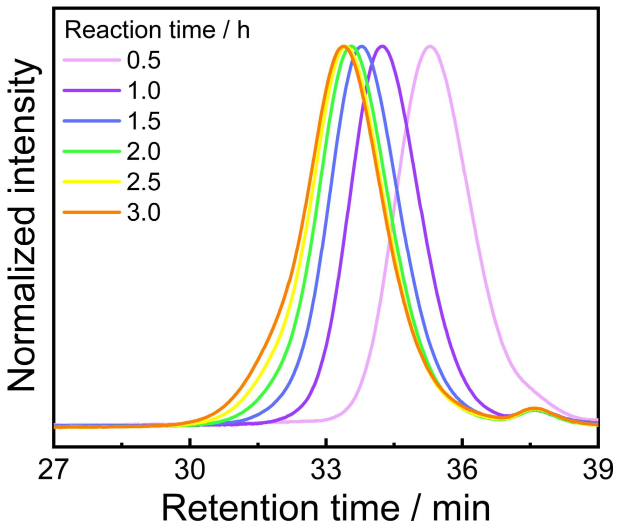

Figure S1. Gel permeation chromatography (GPC) curves of NB-PCL prepared under different polymerization times: 0.5, 1.0, 1.5, 2.0, 2.5, and 3.0 h.

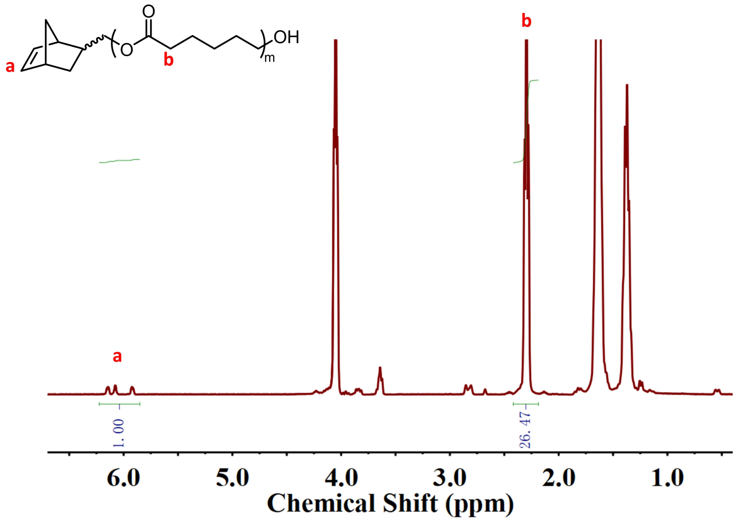

Figure S2. ^{1}H NMR spectrum of NB-PCL in $CDCl_3$.

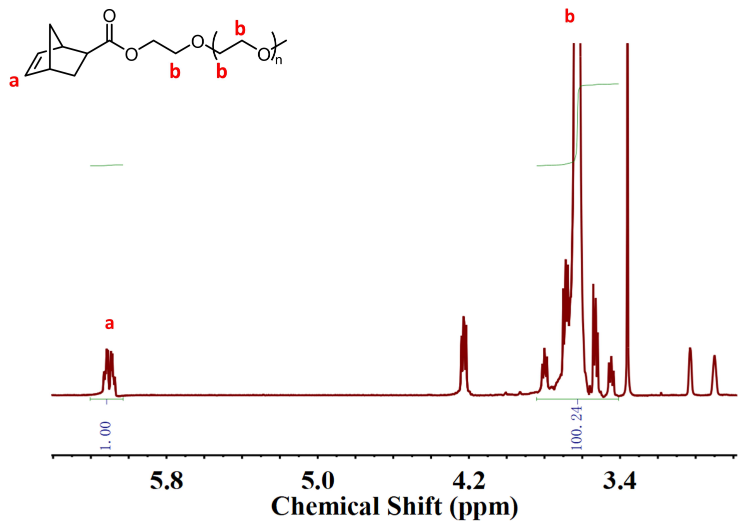

Figure S3. ^{1}H NMR spectrum of NB-PEG in $CDCl_3$.

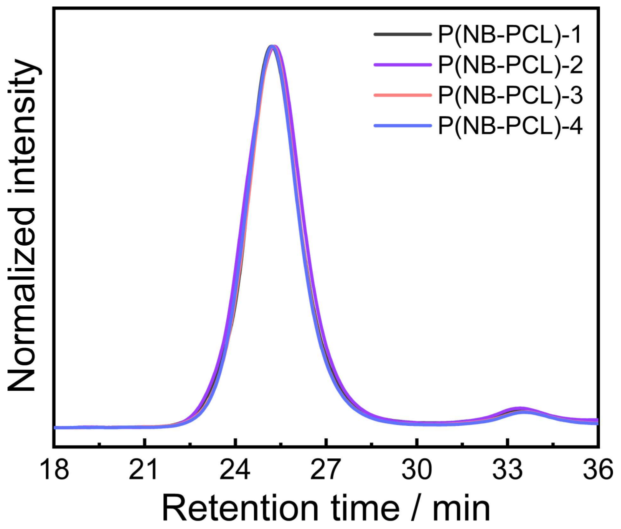

Figure S4. GPC curves of P(NB-PCL) representing four separate synthesis attempts targeting the same composition of P(NB-PCL). These attempts showed very similar GPC curves, with only minor differences, which is indicative of highly repeatable molecular weight and dispersity.

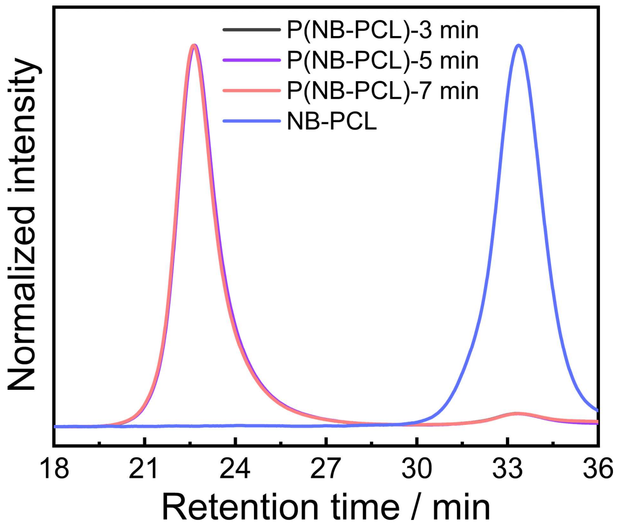

Figure S5. GPC curves of NB-PCL and P(NB-PCL) prepared with different polymerization time: 3, 5, and 7 min.

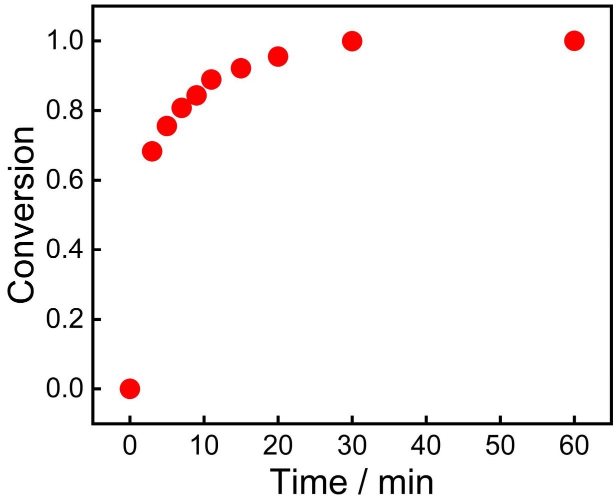

Figure S6. Macromonomer conversion of NB-PEG in the polymerization of poly(NB-PEG) as a function of time, as evaluated by ^{1}H NMR spectroscopy.

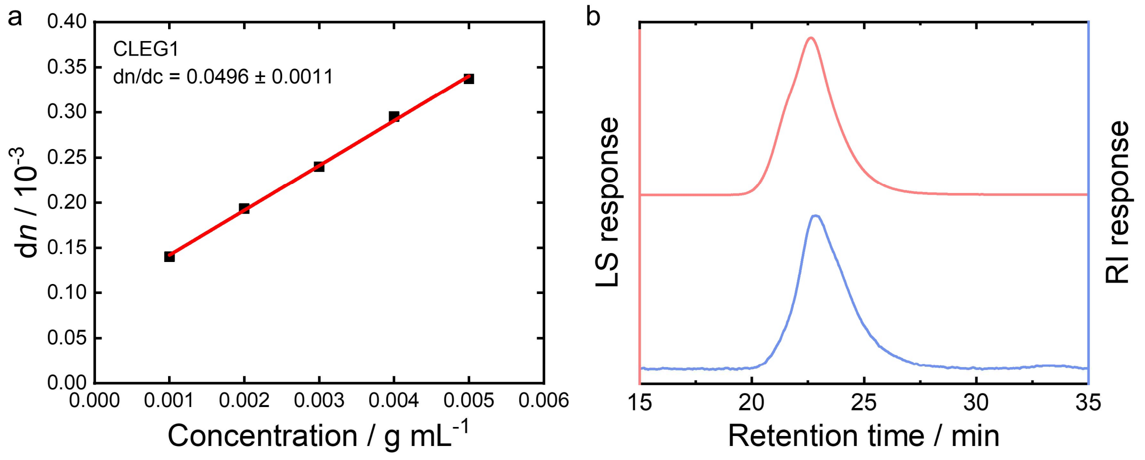

Figure S7. (**a**) Change in refractive index (d*n*) as a function of CLEG1 concentration in DMF. The gradient was subsequently taken as the refractive index increment (d*n*/d*c*). (**b**) Light scattering (LS) and refractive index (RI) signals of CLEG1 as a function of retention time after passing through the GPC columns.

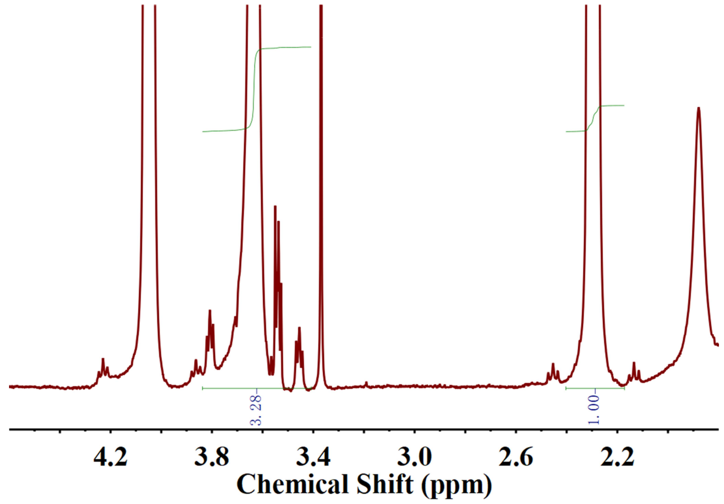

Figure S8. ^{1}H NMR spectrum of CLEG1 in $CDCl_3$.The proton signals from 3.40 to 3.85 ppm correspond to the NB-PEG block, while that from 2.15 to 2.40 ppm correspond to the NB-PCL block. The integral ratio between PEG and PCL is calculated to be 3.28. Combined with the ^{1}H NMR spectra of the NB-PCL and NB-PEG in **Figure S2-3**, the DPs for each block can be obtained as shown in **Table S2**.

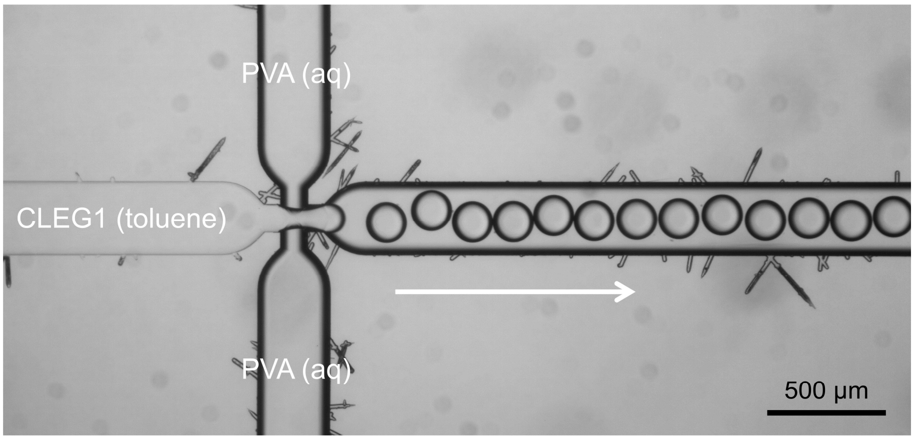

Figure S9. Optical transmission micrograph of the generation of toluene-in-water microdroplets containing 30 mg mL^{-1} CLEG1, using a hydrophilic flow-focusing microfluidic device.

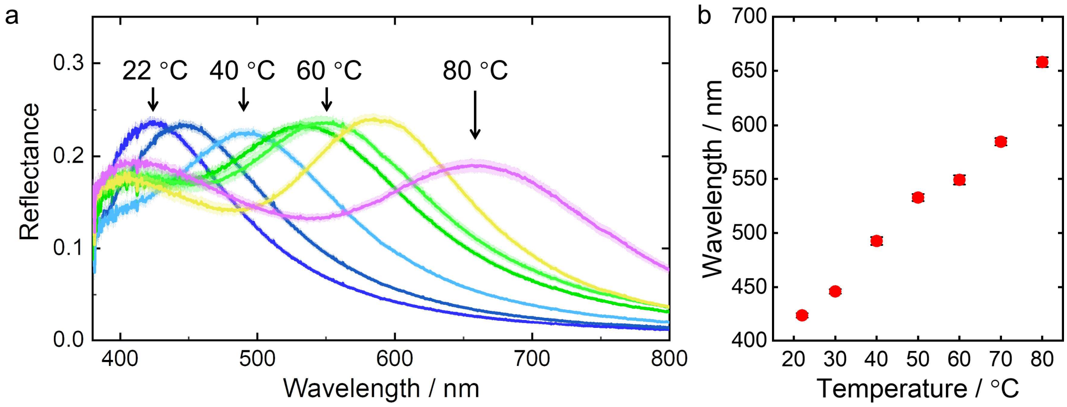

Figure S10. (**a**) Reflectance spectra and (**b**) the corresponding peak wavelengths or the high wavelength peak if there are two peaks in the spectra for the microspheres suspended in water. The intensity was measured relative to a white Lambertian diffuser.

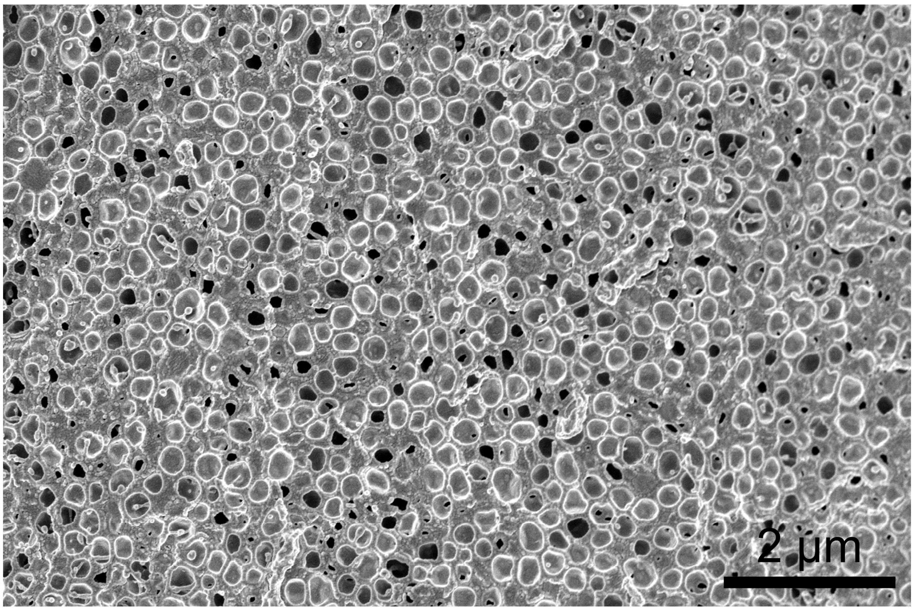

Figure S11. Cryo-SEM image of the sublimated microparticle cross section prepared with CLEG1 at 60 °C. By manually measuring the center-to-center distance, the correlation length was evaluated as 144 ± 18 nm from this image. This value is higher than that measured for the freeze-dried sample (105 ± 4 nm, see Figure 1 and S15), which can be explained by a degree of shrinkage of the entire microparticle during the freeze-drying process. While this shrinkage can underestimate the correlation length and pore size, the high contrast of such images combined with the complete removal of water importantly allows for automated determination of important structural parameters with high precision. In contrast, the sublimation process used in cryo-SEM is complicated and uncontrollable, which can lead to the presence of residual water in the pores, overestimating the filling fraction. Therefore, in this study, we chose to match the experimental spectra directly with the simulated spectra, rather than to simulate the optical response of a reconstructed nanostructure using the parameters from the SEM analysis.

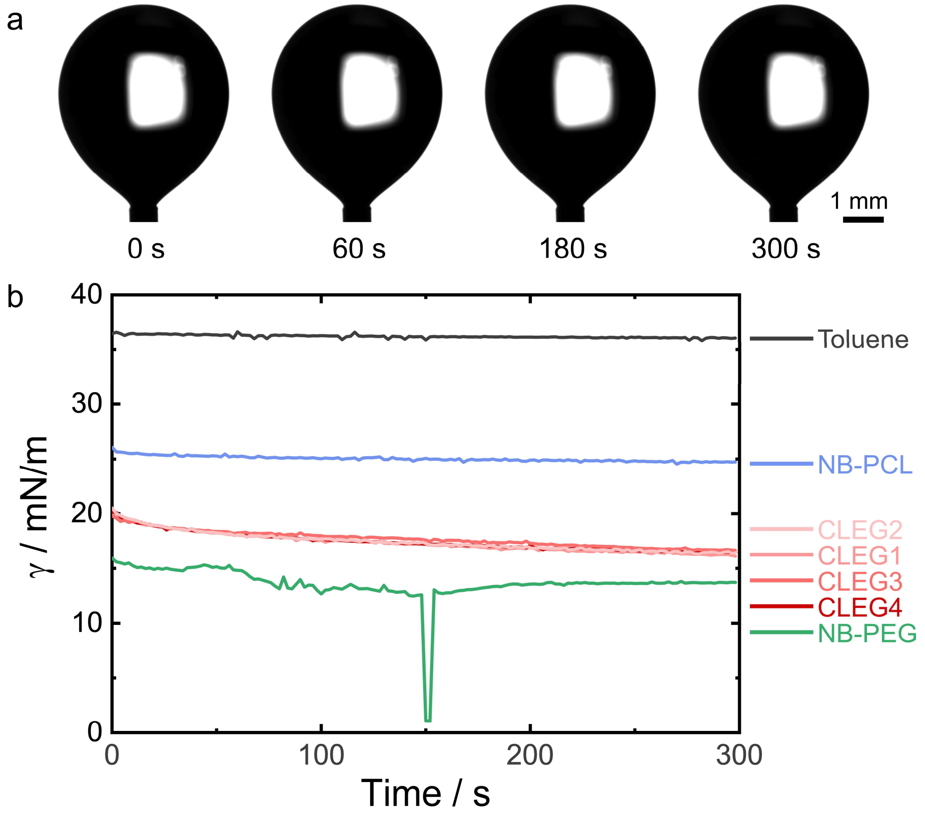

Figure S12. The amphiphilic nature of CLEG1 was assessed via the pendant drop method. (**a**) An inverted toluene drop in water over time, showing only a slight change over the duration of the measurement. (**b**) Interfacial tensions (γ) versus time between water and pure toluene and toluene solutions of NB-PCL, NB-PEG, CLEG1, CLEG2, CLEG3, and CLEG4. The spike in the NB-PEG trace corresponds to a failure to fit the pendant drop for that datapoint. It was found that the dynamic interfacial tension decreased when CLEG1 was introduced, suggesting it can act as a 'giant surfactant'. This surface activity can be accounted for by the hydrophilicity of the PEG block. Notably, when a solution of NB-PEG alone was measured, visible water droplets could be observed to form within the toluene drop (see **Video S1**).

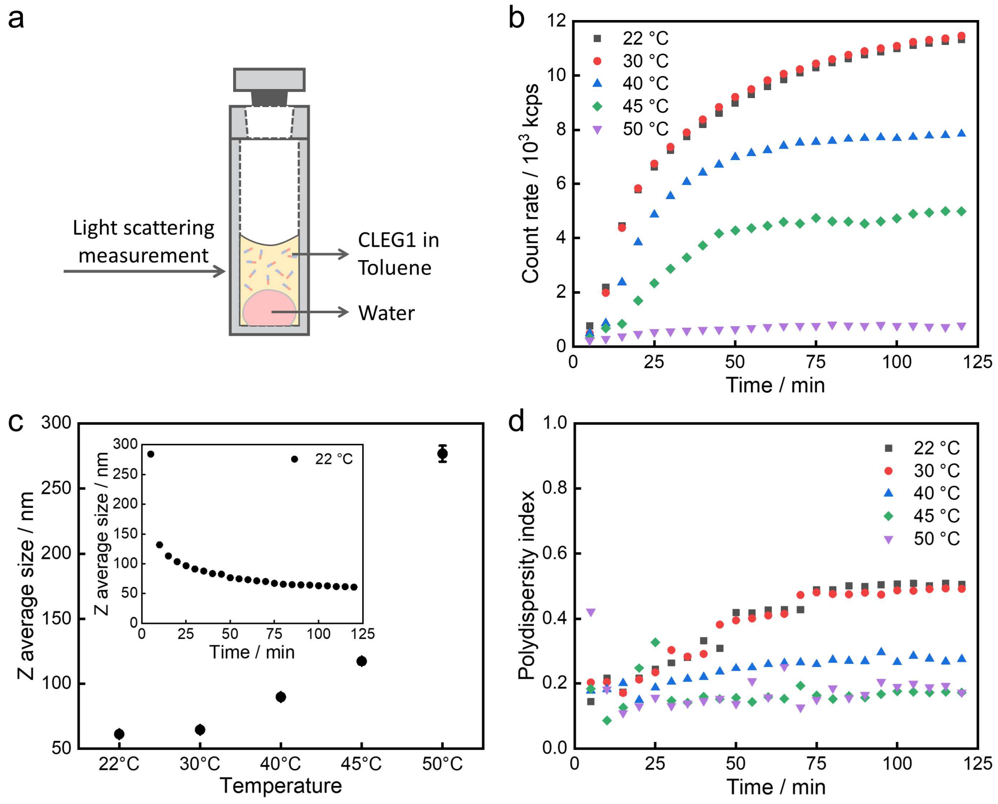

Figure S13. The formation of BBCP micelles can be monitored in real time by dynamic light scattering (DLS). (**a**) The measurement setup used to monitor the formation of micelles when a solution of CLEG1 in toluene is exposed to water. (**b**) Count rate change with time when a droplet of water was added into the CLEG1 toluene solution (5 mg mL^{-1}). In all cases, the count rate appeared to stabilize over time, although the asymptotic value varies with solution temperature. (**c**) Z-average size of the micelles as a function of solution temperature, as measured 120 min after the introduction of water into the polymer toluene solution. The inset displays the time-dependent Z-average size of the micelles formed at 22°C. (**d**) Polydispersity index change of the micelle size as a function of time after water was added to the polymer-toluene solution, for different temperatures. Note that polydispersity index is a number obtained by fitting the correlation data in DLS, which is different from the PDI reported for polymers in **Table S1-2**.

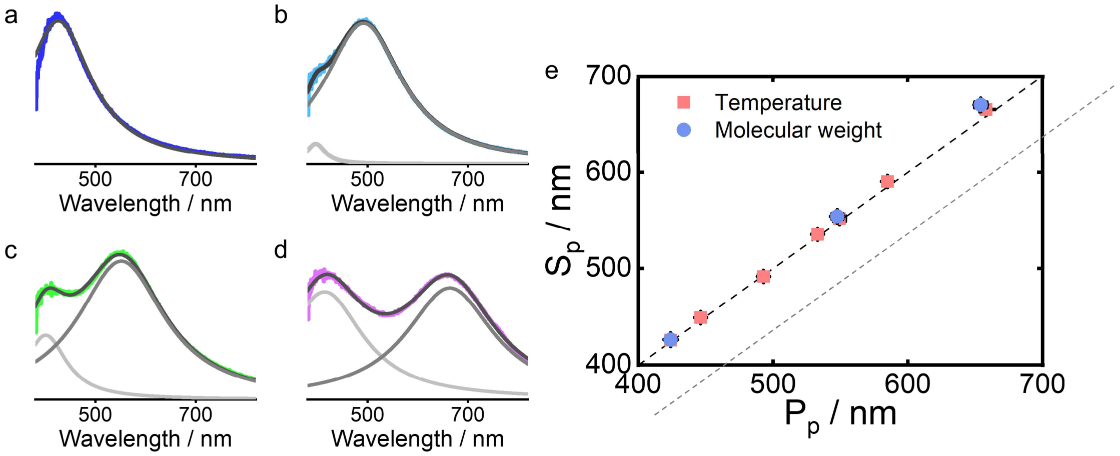

Figure S14. (**a-d**) Reflectance spectra of the samples prepared at (**a**) 22 °C, (**b**) 40 °C, (**c**) 60 °C, and (**d**) 80 °C and deconvoluted by fitting the two peaks with Lorentzian function. Colored curves refer to the measured spectra, while the gray curves are the fitted peaks. The dark grey curves correspond to the first order structural peak and this is the peak referred to within the manuscript, unless otherwise specified. (**e**) Positions of the first order structural peaks (S_p) extracted from the analysis of the fitted spectra, plotted against the values from simple peak picking (P_p), taking the high wavelength peak if there are two peaks in the spectra, see Figure S10.

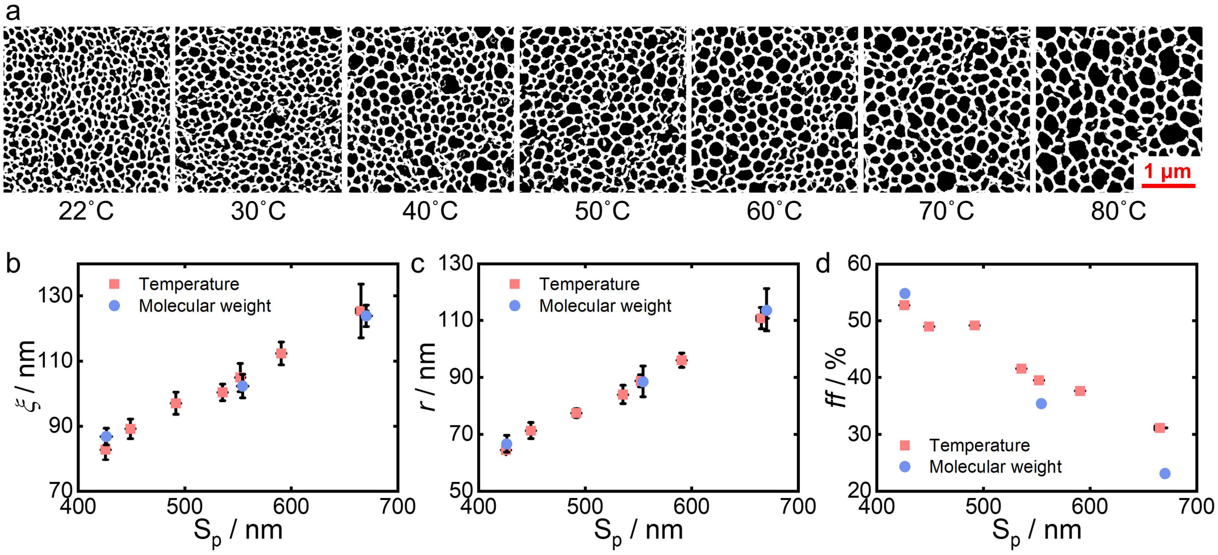

Figure S15. (**a**) Segmented images using ImageJ derived from the SEM micrographs in Figure 1d. (**b-d**) Corresponding structural parameters obtained from the analysis of the segmented images: (**b**) correlation length ξ, (**c**) pore radius r, and (**d**) and filling fraction ff as function of the first order structure factor peak wavelengths S_p.

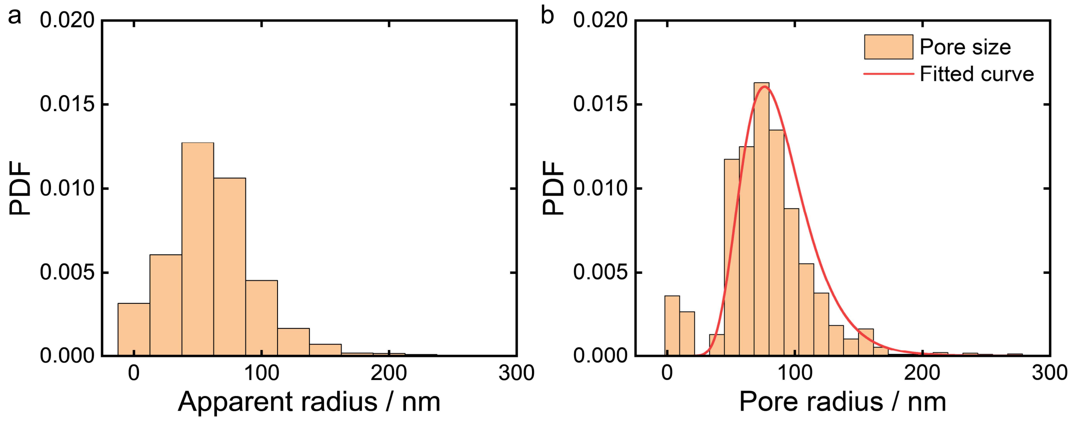

Figure S16. (**a**) Histogram of the apparent pore size extracted from SEM images. (**b**) Histogram and fitted curve of the pore size obtained by Depriester and Kubler based on the Saltykov method.

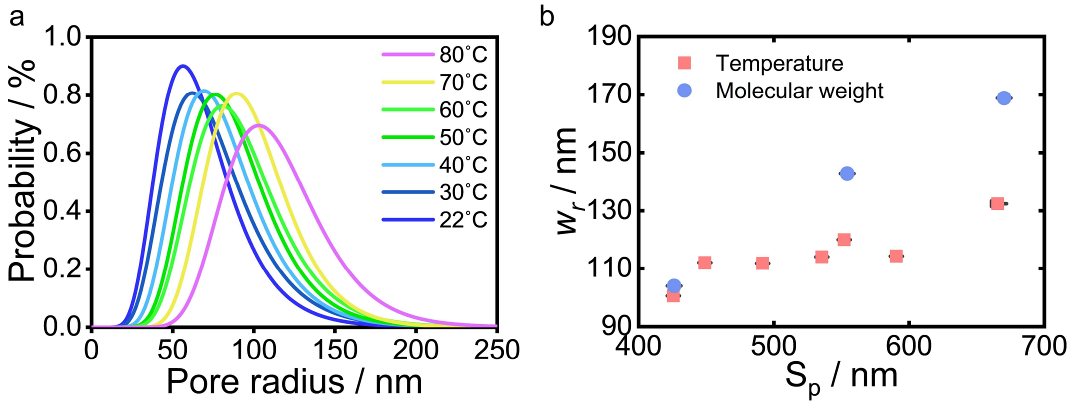

Figure S17. (**a**) Pore radius distribution fitting curves for samples obtained under different temperatures. (**b**) Width of the pore size distribution represented in (a).

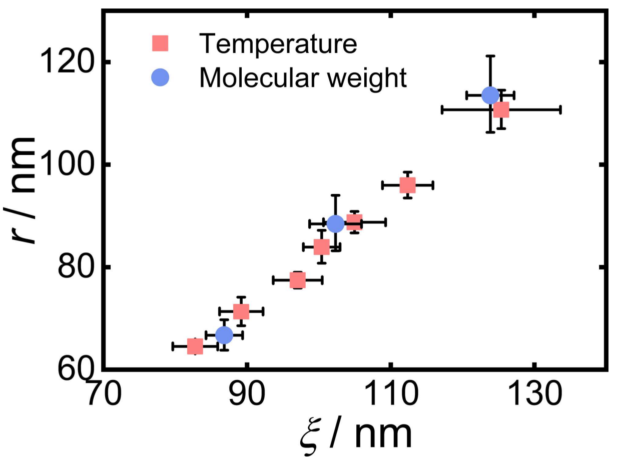

Figure S18. Pore radius versus correlation length from the SEM image analysis in Figure S15.

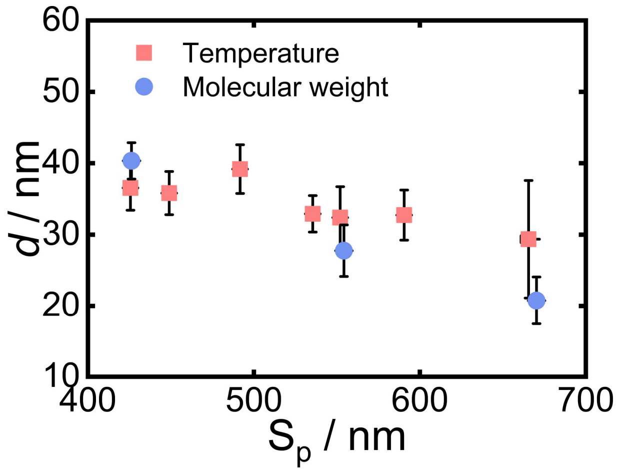

Figure S19. Wall thickness *d* as a function of the first order structural peaks S_p.

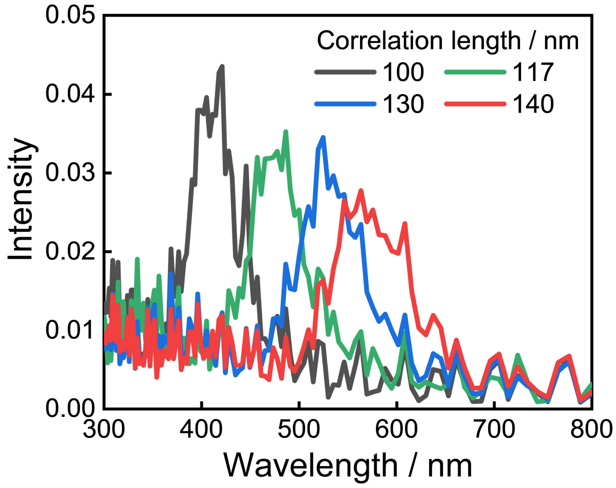

Figure S20. Finite difference time domain (FDTD) simulations on in silico synthesized foam structures with a fixed filling fraction (ff_{sim} = 55%) and increasing correlation length (ξ_{sim} = 100 – 140 nm).

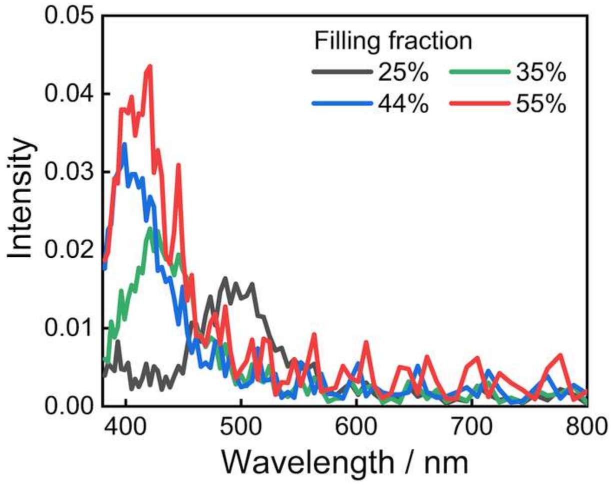

Figure S21. Finite difference time domain (FDTD) simulations on in silico synthesized foam structures with a fixed correlation length (ξ_{sim} = 100 nm) and increasing filling fraction (ff_{sim} = 25 – 55%).

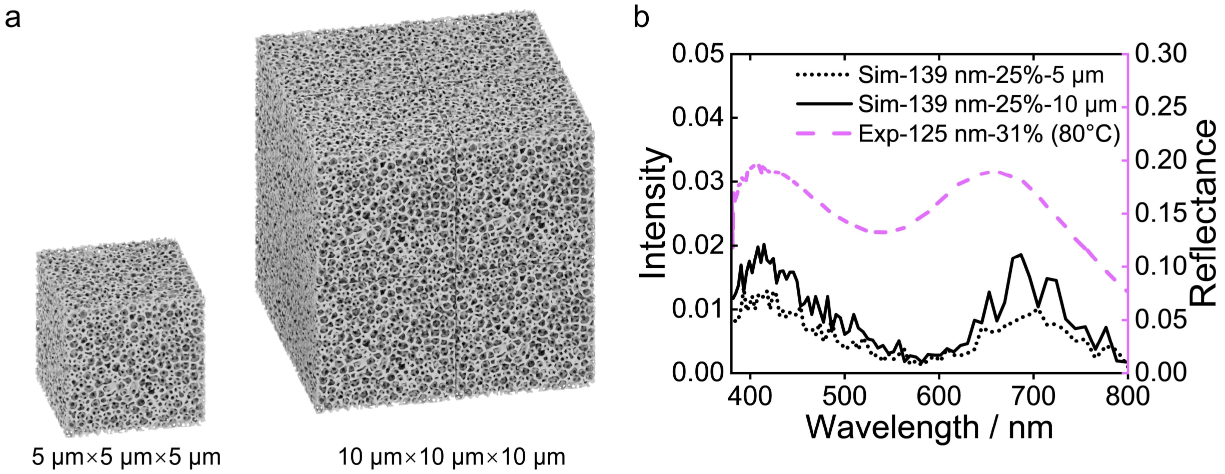

Figure S22. Finite difference time domain (FDTD) simulations on in silico synthesized foam structures at different sizes: (a) foam structures with volumes of 5×5×5 μm and 10×10×10 μm; and (b) their simulated spectra (left axis; black lines) and as a comparison the measured spectrum for photonic pigments prepared at 80 °C (right axis; colored line).

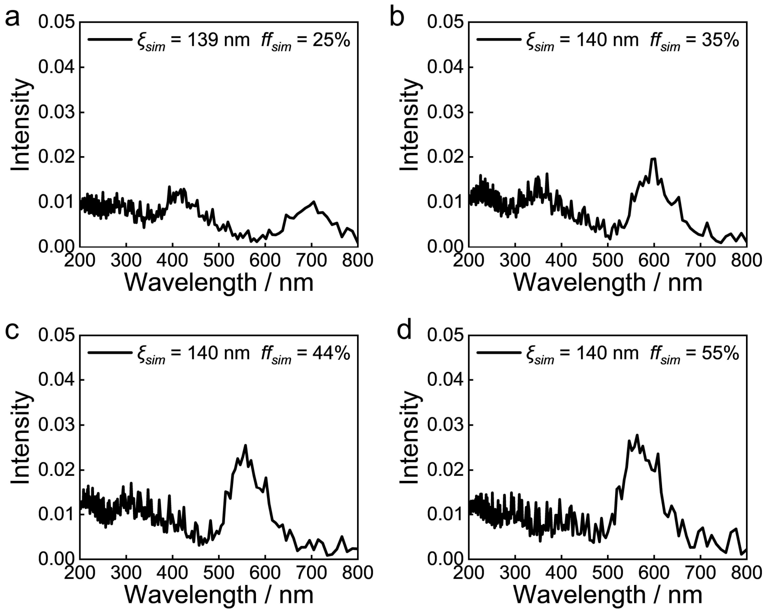

Figure S23. Finite difference time domain (FDTD) simulations on in silico synthesized foam structures with different correlation lengths (ξ_{sim}) and filling fractions (ff_{sim}), denoted ξ_{sim}/ff_{sim} are: (a) 139 nm/25%; (b) 140 nm/35%; (c) 140 nm/44%; (d) 140 nm/55%.

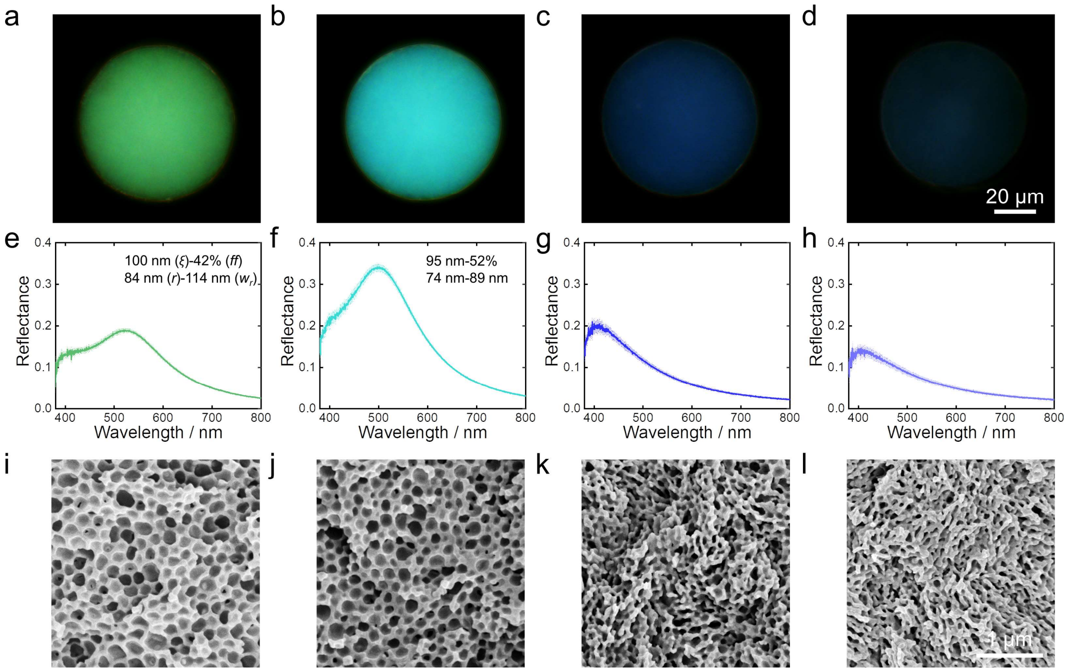

Figure S24. (**a-d**) Micrographs, (**e-h**) reflectance spectra, and (**i-l**) cross-sectional SEM images of photonic pigments prepared with CLEG1 at different conditions: (**a**, **e**, **i**) 50 °C without macromonomer (MM) doping; (**b**, **f**, **j**) 50 °C with 40 wt.% MM; (**c**, **g**, **k**) 50 °C with 80 wt.% MM; (**d**, **h**, **l**) 50 °C with 120 wt.% MM.

As shown in **Figure S24**, a microsphere prepared at 50°C without any MM has a correlation length of 100 nm and a filling fraction of 42%, leading to a green color with a reflection intensity of 0.2 (**Figure S24e**). When 40 wt.% MM relative to the mass of BBCP was blended into the initial toluene solution, (**Figure S24i**, **j**), the filling fraction of the resultant microparticles increases to 52% with only a slight decrease in correlation length (95 nm). As a result, the particle shows a 1.8 fold increase in reflectance to 0.35 in the first structural peak (**Figure S24f**). This increase in reflectance can also be explained by a narrowed pore size distribution which is reflected by decreased w_r from 114 to 89 nm. Further increasing the filling fraction should lead to larger improvements in the color quality, but systematically increasing the amount of MM to 120 wt.% resulted in a deterioration of the short-range order in the porous structure and a large shrinkage in the size of the irregular pores, reducing the correlation length (**Figure S24k-l**). As such, further enhancement of the first structural peak reflectance was not observed.

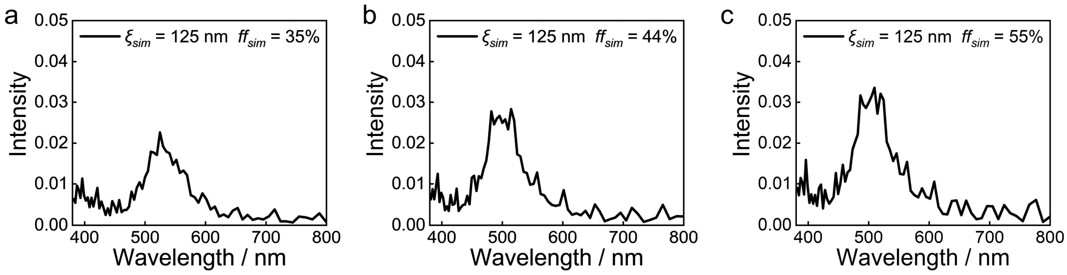

Figure S25. Finite difference time domain (FDTD) simulations on in silico synthesized foam structures with the same correlation lengths (ξ_{sim}) and different filling fractions (ff_{sim}), denoted ξ_{sim}/ff_{sim} are: **(a)** 125 nm/35%; **(b)** 125 nm/44%; **(c)** 125 nm/55%.

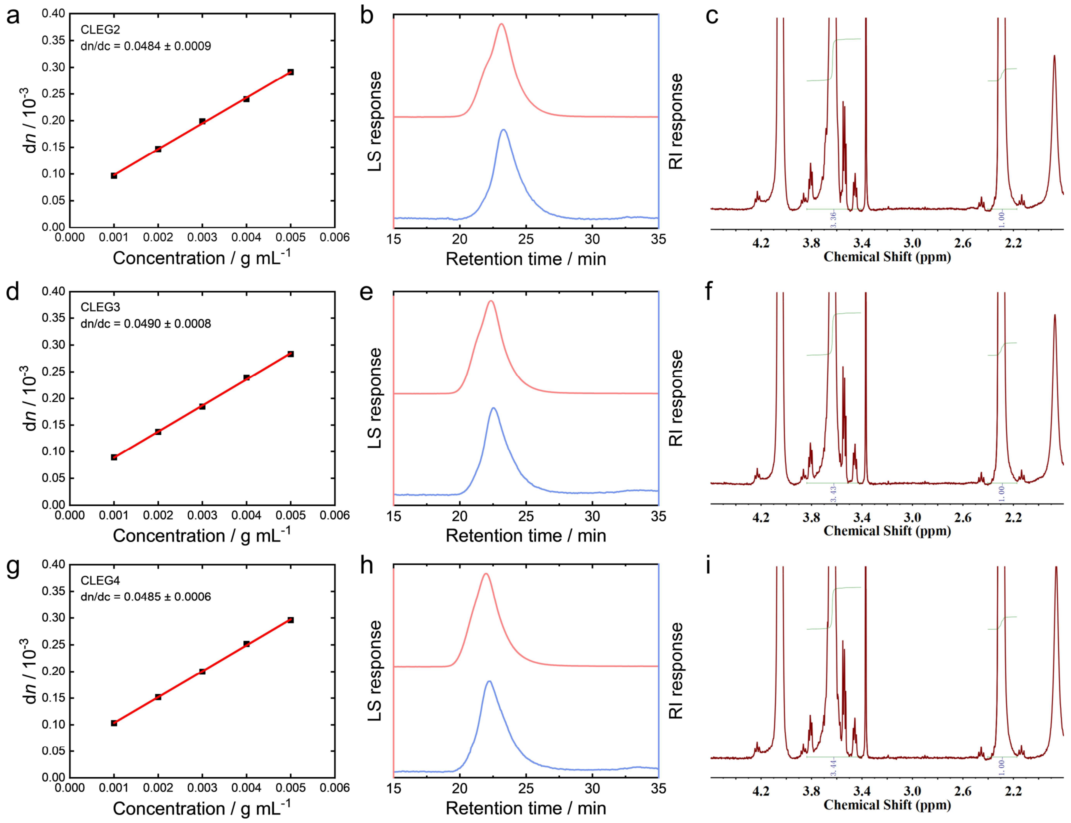

Figure S26. (**a**, **d**, **g**) Change in refractive index d*n* as a function of concentration of CLEG2, CLEG3, and CLEG4 in DMF, respectively. The gradient was taken as the refractive index increment d*n*/d*c*. (**b**, **e**, **h**) LS and RI signals of CLEG2, CLEG3, and CLEG4 respectively as a function of retention time after passing through GPC columns. (**c**, **f**, **i**) ^{1}H NMR spectra of CLEG2-4 in $CDCl_3$. The proton signals from 3.40 to 3.85 ppm correspond to the NB-PEG block, while 2.15 to 2.40 ppm correspond to the NB-PCL block. The NMR integral ratio between PEG and PCL for CLEG2, CLEG3, and CLEG4 are 3.36, 3.43, and 3.44, respectively.

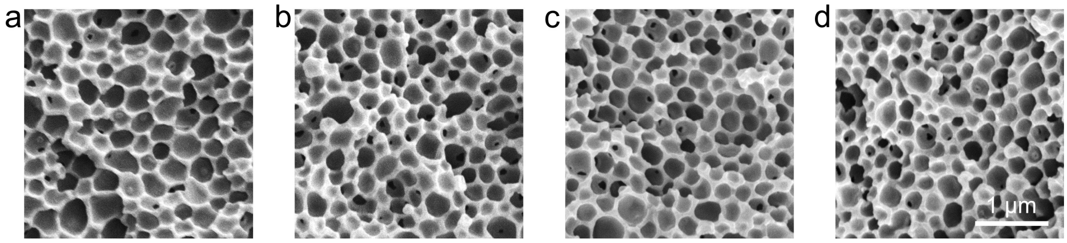

Figure S27. (**a-d**) Cross-sectional SEM images of photonic pigments prepared with CLEG4 at different conditions: (**a**) 60 °C without macromonomer (MM) doping; (**b**) 60 °C with 20 wt.% MM; (**c**) 65 °C with 25 wt.% MM; (**d**) 65 °C with 30 wt.% MM.

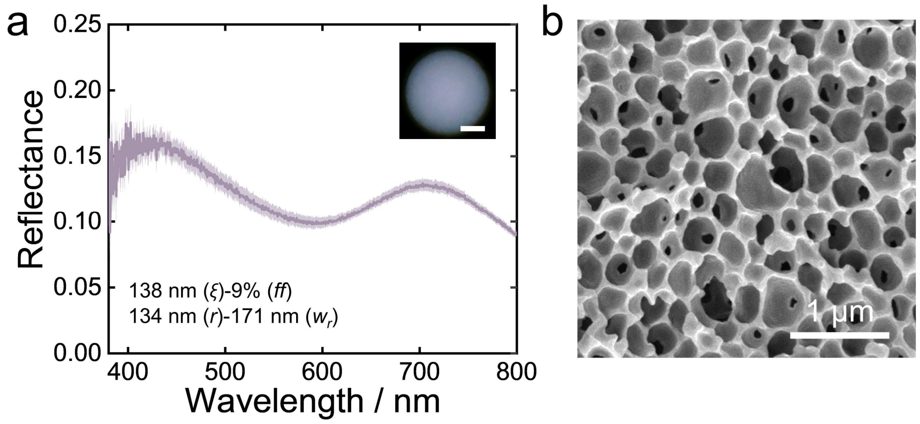

Figure S28. **(a)** Reflectance spectrum and microscopy image and **(b)** cross-sectional SEM image of photonic pigments prepared with CLEG4 at 65 °C. Analysis of the SEM images reveals a correlation length ξ = 138 nm, filling fraction ff = 9%, pore radius r = 134 nm, and pore size distribution w_r = 171 nm. Scale bar for inset is 20 μm.

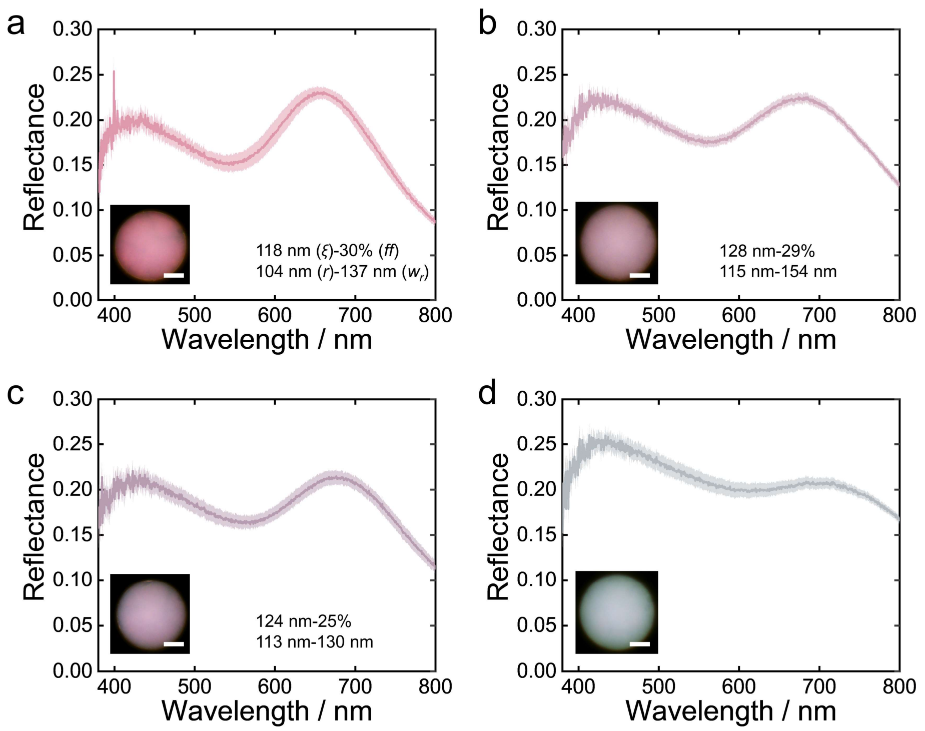

Figure S29. (**a-d**) Reflectance spectra and micrographs of photonic pigments (inset) prepared with CLEG4 at different conditions: (**a**) 70 °C with 30 wt.% MM; (**b**) 70 °C with 35 wt.% MM; (**c**) 75 °C with 30 wt.% MM; (**d**) 75 °C with 35 wt.% MM. In (a-c), the correlation length (ξ), filling fraction (*ff*), pore radius (r), and pore size distribution (w_r) are indicated on the figures and were determined by SEM analysis. For (d), these parameters were not obtained due to failure of the pore size distribution fitting. Scale bar for insets is 20 μm.

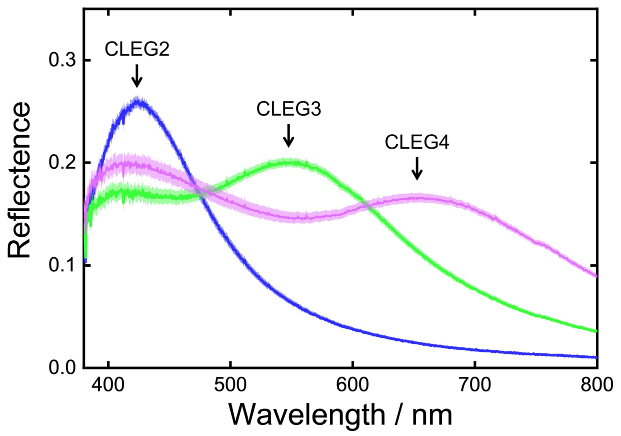

Figure S30. Reflectance spectra of individual photonic pigments prepared with CLEG2, CLEG3, or CLEG4 and dried at 50 °C. The intensity is measured relative to a white Lambertian diffuser.

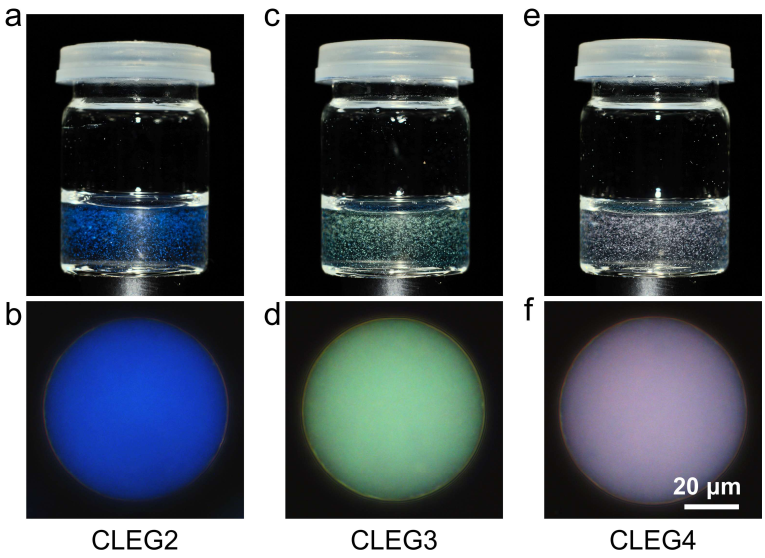

Figure S31. (**a**, **c**, **e**) Photographs of aqueous dispersions of photonic pigments from the self-assembly of (a) CLEG2, (c) CLEG3, and (e) CLEG4 at 50 °C under direct illumination. (**b**, **d**, **f**) Corresponding micrographs of individual microspheres taken from the dispersions shown in (**a**, **c**, **e**).

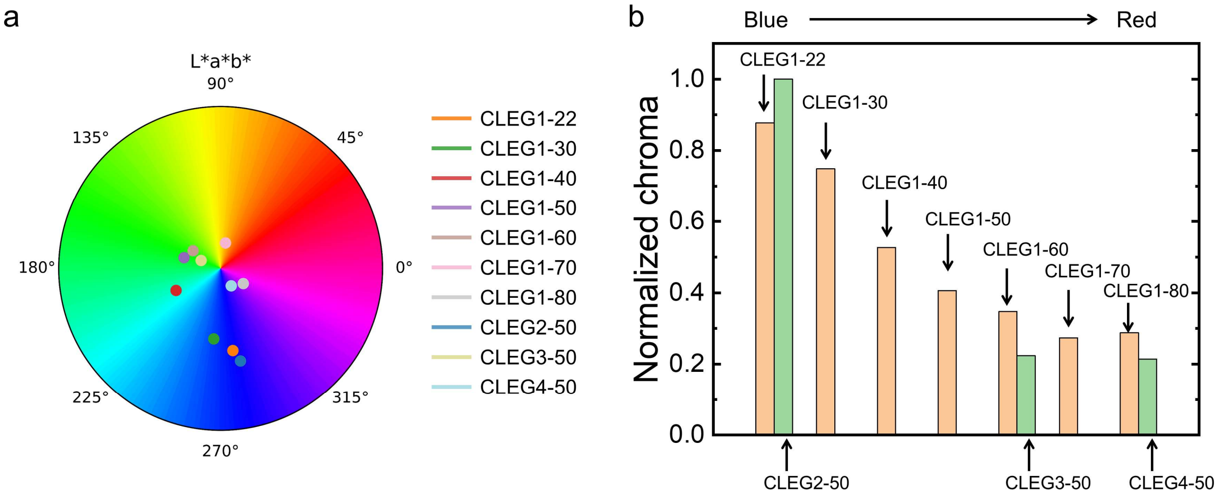

Figure S32. (**a**) CIELAB chromaticity diagram for photonic pigments prepared at different temperatures (T = 22-80 °C) and with different polymers (CLEG1-4). (**b**) The normalized chroma values are calculated according to the method in Section S6. Notably, photonic pigments prepared with CLEG1 at 60 °C and 80 °C have better color purity than for comparably colored pigments respectively prepared from the higher molecular weight CLEG3 and CLEG4 at 50 °C. In addition, the color from the particle obtained with CLEG2 at 50 °C is purer than that from the higher molecular weight CLEG1 at lower temperature (22 °C). This suggests that a single low molecular weight BBCP combined with temperature modulation is a more optimal approach to produce a wide range of colored pigments with good color purity.
